# Femoral Fracture Secondary to a Gunshot Wound Leading to Chronic Expanding Hematoma with Osteomyelitis - An Unusual Presentation of a Pseudotumour: A Case Report

**DOI:** 10.5704/MOJ.2311.012

**Published:** 2023-11

**Authors:** DK Carolino, AR Tud

**Affiliations:** Musculoskeletal Tumor Service, Philippine Orthopedic Center, Quezon City, Philippines

**Keywords:** chronic expanding hematoma, pseudotumour, soft tissue sarcoma

## Abstract

A chronic expanding hematoma (CEH) is a rare clinicopathologic entity that may simulate the clinical and radiologic presentation of soft tissue sarcomas. Etiology has been attributed to repeated exudation and bleeding from capillaries in granulation tissue, resulting in a gradually enlarging mass. A 51-year-old male presented with a large thigh mass following a gunshot wound one year prior. Diagnostic imaging revealed a large complex mass with cystic areas overlying cortical erosions in the femoral diaphysis suggestive of osteomyelitis versus a primary aggressive new growth. Biopsy confirmed CEH and the absence of malignant cells. Hip disarticulation was performed after noting massive necrosis of the thigh compartments and neurovascular compromise. CEH is an important differential diagnosis to be considered in a patient with a slow-growing soft tissue mass and history of significant trauma. Its similar clinical presentation with a soft tissue sarcoma necessitates a high index of suspicion, diagnostic imaging, and biopsy prior to performing definitive surgery.

## Introduction

Chronic expanding hematomas (CEH) were first described as a clinicopathologic entity in 1980 by Reid *et al*^1^. Subsequent accounts of CEH are case reports and small case series, and the authors surmise its rarity may be due to underreporting of such cases. CEH are commonly found in the abdomen and pelvis, while those of the lower extremity are less common^2^ possibly owing to less potential space for expansion.

This poses a diagnostic dilemma due to its similarities to soft tissue sarcomas (STS)^2^. Clinically, both present as a slow-growing mass^2^. On magnetic resonance imaging (MRI), CEH mimic neoplasms by presenting as a central mass of blood surrounded by a wall of dense fibrous tissue^1^. A biopsy is necessary to establish a diagnosis followed by appropriate surgical management^2^.

Here, we present a rare case of a CEH occurring in the thigh of an adult male, initially considered to be a soft tissue sarcoma.

## Case Report

A 51-year-old male sought consult due to a left thigh mass. The patient sustained a gunshot wound to the thigh one year prior, for which he was treated with debridement. Two months after surgery, patient remained non-ambulatory with progressive enlargement of the left thigh which prompted consult.

**Fig 1:**
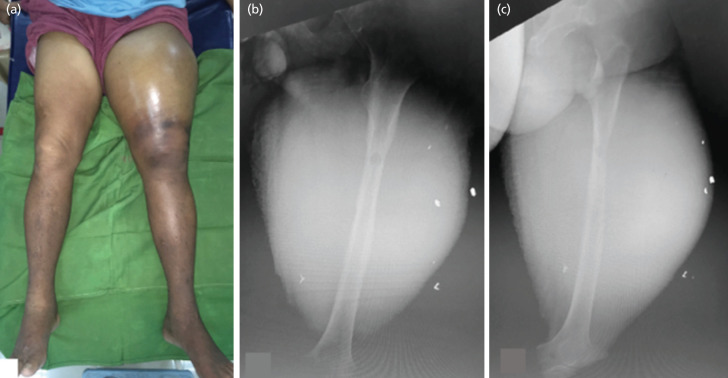
(a) Gross appearance of the bilateral lower extremities. Radiographs of the left femur in (b) anteroposterior and (c) lateral views, show a permeative lytic lesion and cortical erosion in the left femur, surrounded by a large soft tissue mass.

Examination revealed a tense, warm, and tender mass measuring 73.5cm in circumference (Fig. 1a). Left hip and knee range of motion were limited due to severe pain and weakness affecting the entire extremity. The left lower extremity was edematous and distal pulses were weak. On admission, hemoglobin level was at 99 g/L and hematocrit at 0.29. Coagulation profile values were prolonged by 1-2 seconds. Radiographs (Fig. 1b, c) showed lytic lesions and cortical erosion of the femoral shaft, with a large soft tissue mass.

**Fig 2:**
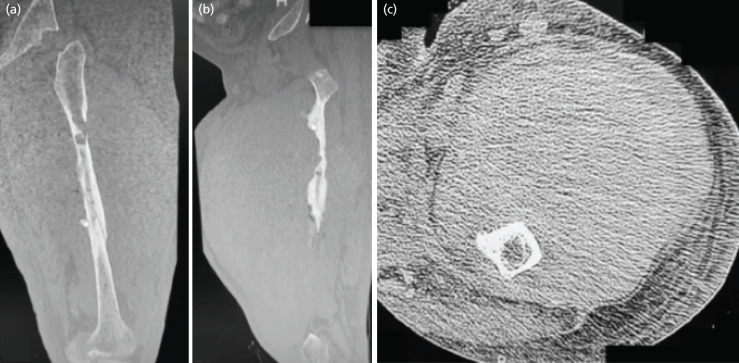
(a) CT scan representative images of the left femur in anteroposterior, (b) sagittal (B), and (c) axial views, noting erosion of the cortex, as well as a large mass on the anterior compartment of the thigh.

Ultrasound revealed a non-compressible mass with areas suggestive of necrosis. Computerised tomography (CT) scans (Fig. 2) showed areas of lysis in the diaphysis, with cortical erosions and an enlarged anterior muscle compartment measuring 34.2 x 16.4 x 13.7cm. Considerations based on imaging were chronic osteomyelitis versus a primary aggressive new growth.

**Fig 3:**
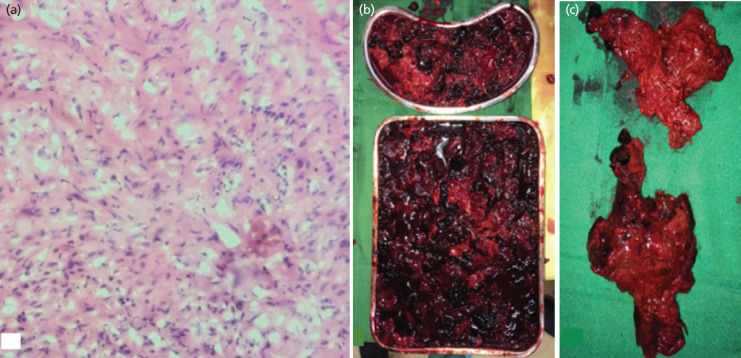
(a) Histologic appearance of specimens, showing blood and fibrin and reactive fibroblastic reaction. Gross appearance of tissue obtained during the surgical procedure, (b) obtaining three litters of hematoma and (c) necrotic muscle fragments.

A sono-guided core needle biopsy revealed necrotic tissues admixed with blood and fibrin. A subsequent open biopsy was consistent with fibroblastic proliferation, chronic osteomyelitis, focal foreign-body giant cell reaction, and no overt evidence of malignancy (Fig. 3a). Patient was thus diagnosed with chronic expanding hematoma and definitive surgery proceeded. Three litres of hematoma (Fig. 3b) and necrotic muscle fragments (Fig. 3c) were then evacuated. Patient was treated with intravenous antibiotics for two weeks and was maintained on drain until with minimal output.

Specimen cultures revealed growth of Escherichia coli, treated with culture-guided antibiotics. Duplex scans showed 50-99% stenosis of the external iliac arteries, as well as acute deep vein thrombosis of the external iliac and common femoral veins. An electrophysiological study of the affected extremity revealed severe axonal lumbosacral (L2-S1) plexopathy.

Due to massive necrosis of the anterior and medial thigh compartment, destruction of the femur, chronic infection, and neurovascular involvement, ablative surgery was recommended. The patient consented and underwent hip disarticulation. Two years post-operatively, the patient is well and without further complaints.

## Discussion

In a series of six patients by Reid *et al* in 1980, five presented with a slowly expanding truncal masses, while only one patient was described as having a mass on the lower extremity^1^. Subsequent reports^2-5^ describing lower extremity CEH consisted of similar patients presenting with soft tissue masses that resembled an STS including synovial sarcoma, epithelioid sarcoma, extraskeletal Ewing sarcoma, and undifferentiated pleomorphic sarcoma. Similarly, this case presented with a gradual enlarging thigh mass which appeared as a soft tissue sarcoma, prompting comprehensive diagnostic examination.

Despite variable locations, gross appearance from all cases consisted of an outer wall of fibrous tissue and a central collection of blood^1^. Histologically, CEH presents with a peripheral zone of collagenous tissue containing hemosiderin-laden macrophages, and multinucleated, foreign body-type, giant cells^1,4^. A midzone has also been observed, containing loose connective tissue fibrin and predominantly amorphous debris, often with capillary ingrowth^1,4^.

An inflammatory cascade is considered in the aetiology of CEH^1^. Bleeding from injury causes hematoma formation^1^. The breakdown of blood within the hematoma activates vasoactive substances that induce capsule formation, while propagating inflammatory cascade resulting in increased vascular permeability and expansion of the hematoma^2–3,5^. Another theory is the presence of vascular endothelial growth factor (VEGF). Activation of VEGF results in angiogenesis, leading to repeated exudation of blood from newly formed capillaries^3,5^. Chronic increased pressure within a closed compartment was theorised to lead to compression of adjacent muscles, impeding blood flow and causing rhabdomyolysis followed by subsequent necrosis^1^.

Evaluation of CEH with CT scans appear multicystic with varied attenuation within the lesion, with or without a partially calcified rim^2,5^. MRI remains the modality of choice and may show a “mosaic sign” which are multiple signal intensities within the lesion corresponding to blood products of different ages^3,5^. Musculoskeletal ultrasound will show a multilocular cystic appearance^5^.

Biopsy with adequate specimens should be done to establish a diagnosis prior to any surgical management. Histologic sections of CEH consist of a mixture of necrotic debris, fibrin, blood clots, cholesterin crystals, and histiocytes, without evidence of malignant cells^4,5^.

While treatment guidelines for CEH are not established, complete surgical excision including the pseudocapsule is the current recommendation of choice^5^. Incomplete excision has been reported to cause further bleeding from a hypervascular subcapsular lesion^5^. Aspiration is not enough to remove the contents of the hematoma and will not remove the fibrous capsule^5^. In this reported case however, the chronicity of disease lead to massive muscle necrosis of the thigh compartments and bony destruction of the femur compounded with osteomyelitis, hence ablative surgery was recommended. This reiterates individualised plan of management for all patients following completion of thorough and complete investigation.

In the presence of a patient with a chronically enlarging extremity mass and a history of trauma, CEH should be considered as a differential diagnosis. With a high index of suspicion, proper imaging modalities can help detect these pseudotumours. Tissue biopsy is necessary to confirm histopathologic diagnosis prior to management.
